# Sex — a potential factor affecting immune checkpoint inhibitor therapy for cancers

**DOI:** 10.3389/fimmu.2022.1024112

**Published:** 2022-10-18

**Authors:** Wang Hu, Xinye Qian, Shuang Wang, Lu Gao, Jingyi Xu, Jun Yan

**Affiliations:** ^1^ Center of Hepatobiliary Pancreatic Disease, Beijing Tsinghua Changgung Hospital, School of Clinical Medicine, Tsinghua University, Beijing, China; ^2^ School of Clinical Medicine, Tsinghua University, Beijing, China; ^3^ Department of Hepatobiliary Surgery, Xuzhou Central Hospital, Xuzhou, China; ^4^ Department of Hepatobiliary Surgery, The No.2 Hospital of Baoding, Baoding, China

**Keywords:** sex, immune checkpoint inhibitor, efficiency, cancer, adverse event

## Introduction

Immune checkpoint inhibitor (ICI) has become one of the most important cancer therapy (1). However, various factors might affect the therapeutic effect of ICI, including tumor micro-environment, systemic immune status, etc. It is believed that the innate and adaptive immune responses of women are higher than those of men. Women could eliminate pathogens faster than men (2); furthermore, about 80% of patients with autoimmune diseases are women (3). This difference in the immune system might affect the natural course of cancer as the mortality caused by various cancers in men is nearly twice than that of women (4). This phenomenon might not only be related to differences in behavioral factors, but also be related to the difference in the immune system between sexes. Animal studies have already shown that sex hormones could regulate the expression and function of PD-1 and PD-L1 (5). Also, the efficacy of anti-PD-L1 mAb was higher in male than that of female mice in the mouse melanoma model (6). As a result, understanding the impact of sex on immune checkpoint inhibitor therapy becomes particularly critical because it might change the strategy for cancer patients of different sexes. Meta analysis could solve unsettled clinical problems. In this article, we explored some of the meta analysis to get a better understanding of the association between sex and ICI therapy among cancer patients.

## Mechanisms of sex affecting immune checkpoint inhibitor therapy

It is speculated that male patients could benefit more from ICI treatment than female patients. There are 3 potential factors for the low efficiency of ICI therapy among female patients.

The first factor is related to the sexual dimorphism of cancer biology. Even after adjusting for age at diagnosis, disease stage, smoking status, and other variables related to tumor mutation load, the tumor mutation burden (TMB) in male patients with multiple types of tumors (including melanoma and non-small cell lung cancer) was significantly higher than that in female patients (7).

The second is the sexual dimorphism of immunity. Women have a stronger immune response than men to lower the mortality of cancer. However, this also means that female tumors must escape from more effective immune surveillance mechanisms and undergo more intensive immune editing processes before metastasis can occur (8). The ability of female tumors to escape immune surveillance might reduce the immunogenicity of advanced female tumors. Moreover, compared with similar tumors in men, the immune escape mechanism is stronger in women. These factors might cause women to be more resistant to immunotherapy. In addition, women’s increased susceptibility to autoimmune diseases might make them more prone to ICI related adverse events, resulting in a higher rate of treatment discontinuation (8).

The third is the sexual dimorphism of hormones and their receptors. Estradiol, *via* estrogen receptorα, induces the polarization of tumor associated macrophages (TAMs) toward the immune-suppressive M2 phenotype at the expense of the anti-tumor M1 phenotype, leading to a dysfunctional cytotoxic T cell antitumor response (9). This might also damage the treatment effect of ICI in women patients.

## Adverse event of ICI therapy between sexes in clinics

Unger et al. (10) explored the sex difference of immunotherapy and other therapies to evaluate the risk difference of serious adverse events between women and men among different treatment methods, including ICI therapy.

The investigators included 27 specific adverse events from 202 studies, including 13 symptomatic adverse events and 14 objective adverse events. Of all 23,296 patients, 2,319 received immunotherapy. Compared with men, all women had a 34% increased risk of serious adverse events (68.6% *vs*. 62.2%, P < 0.001). An increased risk of severe toxicity was observed in women in each treatment regimen, with the greatest difference in toxicity risk among immunotherapy (33.7% in women *vs*. 25.4% in men, P < 0.001). The association was strongest between adverse events women receiving targeted therapy or immunotherapy (OR 1.42, P < 0.001).

This study showed that among various treatment regimens, women had a higher risk of serious symptomatic adverse events, and the risk of symptomatic adverse events in women receiving immunotherapy was 66% higher than that in men. In addition, women receiving immunotherapy were more likely to have serious hematological adverse events. All these results indicated that ICI therapy differ between women and men.

## Overall survival of ICI therapy between sexes in clinics

Conforti et al. (11) first explored overall survival (OS) between women and men receiving ICI by meta-analysis, which included 20 randomized controlled trials. Among them, 13 involved PD-1 inhibitors, six involved CTLA-4 inhibitors, and one involved PD-1 inhibitors combined with CTLA-4 inhibitors. There were seven trials for the treatment of melanoma, six for non-small cell lung cancer, two for head and neck cancer, one for small cell lung cancer, one for renal cell cancer, one for urethral tumor, one for gastric cancer and one for mesothelioma. eight trials were first-line treatment, and 12 were second-line or rear line treatment.

A total of 11,351 patients were included in this analysis, including 7,646 males (67%) and 3,705 females (33%). Male patients who received ICI therapy had a significantly lower risk of death than those who received control drug treatment. In female patients, ICI benefits were smaller compared with controls. Further, ICI efficacy was higher in male than in female (P = 0.0019).

However, Graham et al. (12) analyzed the data of patients with metastatic renal cell carcinoma treated with nabulizumab in the international joint database of metastatic renal cell carcinoma. A total of 294 patients received nabulizumab and 1463 patients received everolimus. The authors found that the OS outcome of navarizumab was better than that of everolimus, but sex had no effect on the efficacy of navarizumab *vs*. everolimus.

Wallis et al. (13) conducted a meta-analysis including 23 trials, finding that compared with other cancer therapies, the OS advantage of patients in the ICI treatment group was statistically significant. Compared with standard cancer therapy, OS benefit of ICI treatment was observed in both male and female patients, and there was no significant difference in OS advantage between male and female patients (P = 0.60).

According to these results, it seems that sex would not affect the efficiency of ICI therapy. Yet, these two studies are based on the subgroup risk ratio (HR) of published clinical trials. They are lack of analysis of individual patients while some clinical characteristics (including smoking behavior and clinicopathological subtypes) are distributed differently between men and women, which might cause bias.

Ye et al. (14) adopted an innovative method to calculate the trial specific HR ratio and applied a random effects model to calculate the pooled HR. After combining 27 clinical trials, it was found that six of the eleven trials showed the OS advantage of male patients, while the other five showed the OS advantage of female patients.

To sum up, it is still difficult to solve the debate whether sex is related to the efficacy of ICI simply by meta-analysis. Prospective clinical studies might be needed in the future to clarify the answer.

## Prospective

It is important to establish an association between sex and anti-cancer immunity through further research. After all, it is easy to use hormone therapy to affect the efficacy of immunotherapy. Preliminary data suggest that blocking the androgen axis by androgen deprivation therapy (ADT) in combination with enzalutamide has the potential to reverse the resistance of tumors other than castration resistant prostate cancer to PD (L) 1 monoclonal antibody monotherapy and improve the activity of other immunotherapy regimens, such as adoptive T-cell therapy (15). It is important to evaluate the impact of controlling the androgen and estrogen axis in various conditions to understand the potential heterogeneity under different immunotherapy strategies, tumor types, patient age, and menopausal status.

Unfortunately, so far, host related factors (especially patient gender) have been largely ignored in preclinical and clinical studies of anti-tumor immunotherapy. Relatively few women were included in ICI RCTs. The low proportion of female participants could be seen in trials of different types of tumors, regardless of whether there is sex difference in tumor incidence rate. More seriously, only a very few trials used patient sex as a stratification factor in the design. Similar bias also affected preclinical studies. In cancer cell line Encyclopedia (CCLE), a cancer cell line database of Broad Institute, the proportion of male and female cell lines is unbalanced to a large extent. There are more male cell lines than female cell lines in many tumor types. Some tumor types even have no female cell lines (such as bile duct tumors and salivary gland tumors).

Moreover, most mouse experiments only used young premenopausal female mice. Although sex differences in cancer susceptibility in mice are well known, we still lack in-depth understanding of them. The results of preclinical studies including only male animals and clinical studies including only male participants do not necessarily apply to female patients. An in-depth understanding of the role played by gender in immunity and treatment will help us gain new knowledge about biological and therapeutic targets, thereby improving the prognosis of female and male cancer patients.

## Author contributions

WH, XQ, and SW conceived of the project. WH, XQ, SW, LG, and JX collected and analyzed the data. WH, XQ, and SW wrote the paper. JY provided expert guidance and suggestions. All authors contributed to the article and approved the submitted version.

## Funding

Start up Fund for Talent Researchers of Tsinghua University (No. 10001020507).

## Conflict of interest

The authors declare that the research was conducted in the absence of any commercial or financial relationships that could be construed as a potential conflict of interest.

## Publisher’s note

All claims expressed in this article are solely those of the authors and do not necessarily represent those of their affiliated organizations, or those of the publisher, the editors and the reviewers. Any product that may be evaluated in this article, or claim that may be made by its manufacturer, is not guaranteed or endorsed by the publisher.
